# PolyLLM: polypharmacy side effect prediction via LLM-based SMILES encodings

**DOI:** 10.3389/fphar.2025.1617142

**Published:** 2025-07-31

**Authors:** Sadra Hakim, Alioune Ngom

**Affiliations:** School of Computer Science, University of Windsor, Windsor, ON, Canada

**Keywords:** drug combination, large language models, polypharmacy side effect, smiles, graph neural networks

## Abstract

Polypharmacy, the concurrent use of multiple drugs, is a common approach to treating patients with complex diseases or multiple conditions. Although consuming a combination of drugs can be beneficial in some cases, it can lead to unintended drug-drug interactions (DDI) and increase the risk of adverse side effects. Predicting these adverse side effects using state-of-the-art models like Large Language Models (LLMs) can greatly assist clinicians. In this study, we assess the impact of using different LLMs to predict polypharmacy. First, the chemical structure of drugs is vectorized using several LLMs such as ChemBERTa, GPT, etc., and are then combined to obtain a single representation for each drug pair. The drug pair representation is then fed into two separate models including a Multilayer Perceptron (MLP) and a Graph Neural Network (GNN) to predict the side effects. Our experimental evaluations show that integrating the embeddings of Deepchem ChemBERTa with the GNN architecture yields more effective results than other methods. Additionally, we demonstrated that utilizing complex models like LLMs to predict polypharmacy side effects using only chemical structures of drugs can be highly effective, even without incorporating other entities such as proteins or cell lines, which is particularly advantageous in scenarios where these entities are not available.

## 1 Introduction

Polypharmacy, commonly defined as the concurrent use of multiple medications by the same patient, has become increasingly prevalent, particularly among older adults with multimorbidity Masnoon et al. (2017); Joaquim and Campos (2011). This is because multiple medications are often required to manage different health conditions, especially in elderly patients, to ensure effective treatment. When prescribed appropriately, polypharmacy can improve health outcomes and enhance quality of life. For instance, studies have shown that drug combination with synergistic effects can increase the success rates of drug repositioning Sun et al. (2016); Madani Tonekaboni et al. (2018).

However, polypharmacy is also associated with significant risks Srivastava (2024); Plácido et al. (2021). Research indicates that polypharmacy increases the likelihood of adverse drug reactions (ADRs) and drug-drug interactions (DDI) due to complex medication regimens Srivastava (2024). The risk of DDIs rises as the number of medications increases, consequently heightening the potential for negative health outcomes Maher et al. (2014). A recent study examining trends over 20 years, from 1999 to 2018, in the United States found that polypharmacy is continually increasing, particularly among older adults and patients with heart disease or diabetes Wang et al. (2023).

With the growing application of deep learning in various domains, including drug-related research, we aim to utilize Large Language Models (LLMs) and traditional language models to predict the side effects of drug combinations. In this study, we retrieved a chemical representation of drugs known as the Simplified Molecular Input Line-Entry System (SMILES) Weininger (1988) from PubChem Kim et al. (2016). After encoding these SMILES strings and obtaining representations for drug pairs, the resulting features are fed into two distinct classifiers, including a Multilayer Perceptron (MLP) and a Graph Neural Network (GNN), to evaluate and compare their performance in predicting side effects.

LLMs have revolutionized natural language processing (NLP) by achieving remarkable success in general tasks. However, their application in scientific disciplines, particularly chemistry, is still in its early stages. This presents an opportunity for significant advancements in this area. In particular, researchers have begun integrating LLMs within the context of drug discovery. For example, Sadeghi et al. (2024) compared the performance of Generative Pre-trained Transformer (GPT) Radford et al. (2019) and Large Language Model Meta AI (LLaMA) Touvron et al. (2023a); Touvron et al. (2023b) with pre-trained models on SMILES, such as ChemBERTa Chithrananda et al. (2020), in vectorizing SMILES strings, on two downstream tasks, including molecular property prediction and drug-drug interaction prediction. Similarly, Pham et al. (2024) demonstrated the superior performance of ChemBERTa Chithrananda et al. (2020) compared to a model which constrimilarity profiles by comparing Morgan fingerprints Rogers and Hahn (2010) in predicting the clinical relevance of interactions between a pair of drugs. Furthermore, Xu et al. (2023) used Simple Contrastive Sentence Embeddings (SimCSE) Gao et al. (2021) to fine-tune the original Bidirectional Encoder Representations from Transformers (BERT) Devlin et al. (2018) model to encode drugs and integrate them with cell line features for predicting synergistic drug combinations.

Despite these advancements, there is still a lack of research on the impact of LLMs in encoding drugs and comparing their performance. Several studies, such as Masumshah and Eslahchi (2023), have approached this task by forming binary vectors for each drug feature and then computing Jaccard similarity between drug pairs for the specific features and then being aggregated to obtain a single representation per drug. The DeepPSE model Lin et al. (2022) takes a different approach by using deep learning and self-attention mechanism. It constructs drug representations by concantenating mono side effect features and drug-protein (DPI) features. Then, multiple neural networks, including Convolutional Neural Networks, autoencoders with self-attention mechanism, and a Siamese network are used to generate drug pair representations. These representations are fused and passed through fully connected layers to predict side effects. Another study Masumshah et al. (2021) applied Principle Components Analysis (PCA) over binary feature vectors of single drug side effects and drug-protein interactions to obtain a representation for the corresponding drug. After summing the representations of drug pairs, an MLP is employed to classify the drug pair into its associated side effects.

Several studies have used graph neural networks for drug-related predictions. For instance, Wang et al. (2020) proposed a method based on an enhanced domain knowledge graph that integrates drugs, genes, and enzymes, and while it captures various types of interactions. After encoding these relationships by generating entity embeddings and concatenating head and tail entities, they apply a Convolutional Neural Network (CNN), followed by fully connected layers, to compute interaction scores. Additionally, a recent study by Cheng et al. (2024) introduced HANSynergy to predict drug synergies. They constructed a heterogeneous graph that captures diverse relationships associated with drug combinations, including protein-drug, drug-cell line, protein-protein, protein-cell line, and tissue-cell line interactions. To extract features, they use SimCSE model and RDKit tolls for drug SMILES and proteins sequences, while using word2vec and gene expression profiles for cell lines and tissues. After training the graph, the extracted features were fed into a multilayer perceptron to predict whether a drug combination is synergistic or antagonistic.

A significant challenge in existing methodologies is their dependence on supplementary drug-related features, such as proteins, cell lines, enzymes, or gene profiles, which are often limited or unavailable. Additionally, most current studies predominantly utilize one-hot encoding methods and similarity computations to represent entities, resulting in embeddings that may lack the depth and informativeness provided by advanced pre-trained language models. To address these limitations, this study focuses explicitly on the chemical interactions between drugs, rather than incorporating additional biological entities. By leveraging sophisticated language models to generate more informative and rich drug embeddings, we demonstrate that our proposed model achieves superior performance compared to previous approaches, while significantly reducing the reliance on extraneous features. Consequently, our model shows greater generalizability and resource efficiency, effectively overcoming the constraint of feature unavailability.

## 2 Methodology

### 2.1 Dataset

In this study, we use the Decagon dataset, the sole preprocessed resource available for drug-drug-side effect analysis. This dataset is originally introduced in the Decagon paper Zitnik et al. (2018). It includes both drug-drug interactions and their associated polypharmacy side effects. Drug-drug interactions typically occur when one drug alters the effects of another drug, potentially leading to adverse outcomes. The dataset’s polypharmacy side effect information within the dataset is sourced from the TWOSIDES dataset Tatonetti et al. (2012), and contains 4,649,441 drug pair-side effect associations, 1,318 side effect types across 645 drugs and 63,473 distinct drug combinations. The side effects in the TWOSIDES dataset are extracted from the U.S. Food and Drug Administration’s (FDA) Adverse Event Reporting System (AER). The Decagon dataset was preprocessed to use standardized side effect terms with synonyms removed, ensuring a controlled and consistent vocabulary.

### 2.2 Data preparation

The decagon dataset serves as a comprehensive resource, providing detailed information for each drug, including its Compound ID (CID), and the Concept Unique Identifier (CUI) for the associated polypharmacy side effects, alongside descriptive names of these side effects. We aim to employ text-based molecular representations of chemical structures and encode them using advanced language models. To achieve this, we use PubChem Kim et al. (2016), a publicly accessible repository that contains extensive chemical substance data and biological activity profiles. PubChem organizes this vast amount of information into three interconnected databases with unique identifiers: Substances (SID), Compound (CID), and BioAssay (AID).

PubChem provides one unique SMILES string per CID for a drug, and ensures that the structure is represented consistently. This is achieved through an internal algorithm that ensures the SMILES string is deterministic and canonicalized. Using the CID of the drugs within our dataset, we retrieve detailed chemical information for each drug from PubChem, specifically extracting their standardized SMILES (Simplified Molecular Input Line Entry System) strings. Consequently, the modified dataset now consists of Drug-1 SMILES, Drug-2 SMILES, and the corresponding Side Effect Name, establishing a structured foundation for predictive modeling.

Molecular representation can take various forms, such as text-based, structure-based, and feature-based formats. In this study, we focus on the SMILES format, which is a concise linear string representation that encodes atoms, bonds, and branching in molecular structures. The character-level representation of SMILES facilitates straightforward tokenization, making it an ideal input for language models Janakarajan et al. (2023). An alternative text-based format, SELFIES (Self-referencing Embedded Strings) Krenn et al. (2020), addresses some limitations of SMILES. While generating SMILES strings can pose challenges, such as potential invalidity due to an uneven number of ring openingclosing symbols or bond valence violations Janakarajan et al. (2023), recent studies Skinnider et al. (2021) have shown that language models can effectively distinguish between valid and invalid molecule representations by learning the syntax of SMILES, thereby enhancing their utility for cheminformatics applications.

To align our approach with prior studies, we focus on predicting 964 commonly occurring types of polypharmacy side effects, each present in at least 500 drug combinations. This selection reduces the total number of drug combinations to 4,576,785, ensuring comparability with established baselines.

To verify the structural uniqueness of these 645 drugs, we further processed the canonical SMILES strings by converting them to full InChIKeys using RDKit. The InChIKey is a hashed representation of the complete molecular structure, derived from the International Chemical Identifier (InChI), which encodes atom connectivity, hydrogen positions, and salt components. In other words, InChI distinguishes between different forms of the same compound, such as between tautomers and between different salt forms. In our analysis, all 645 SMILES strings produced unique full InChIKeys, confirming that no duplicate or structurally redundant compounds were present in the dataset.

In this dataset, a single drug pair can be associated with multiple side effects, meaning the problem cannot be treated as a multi-class classification task. The Decagon dataset initially records each side effect of a drug pair as a separate entry. To prepare the data for input into the multilayer perceptron, we integrated these entries by grouping all side effects for each drug pair into a single list. For instance, if Drug-1 and Drug-2 have side effects A,B , and C listed in separate rows, we combine these into a unified list $\left[A,B,C\right]$ , resulting in a single row per drug pair. This transformation reframes the problem as a multi-label classification task, where each drug pair is associated with a set of side effects. As a result, the dataset size is reduced from 4,576,785 rows to 63,472 rows, significantly simplifying further analysis and optimizing its manageability.

Additionally, we encode the list of side effects into a multi-hot vector format to facilitate input for the multilayer perceptron approach. This process involves converting side effect names into numerical representations. Specifically, we construct a binary vector of size 964 for each drug pair, where each element in the vector indicates the presence (1) or absence (0) of a specific side effect. This transformation enables efficient handling of the multi-label classification problem.

### 2.3 Data analysis

Following the completion of dataset preprocessing, we conducted an analysis to gain deeper insights into the model configuration requirements. When tokenizing sequences using LLMs such as BERT, it is crucial to specify an appropriate maximum input length to ensure uniform processing of inputs by the model. This parameter is vital, as setting the maximum length too low may result in the truncation of essential information from longer sequences, while setting it too high can introduce excessive padding, leading to computational inefficiency and a potential loss of accuracy.

**FIGURE 1:**
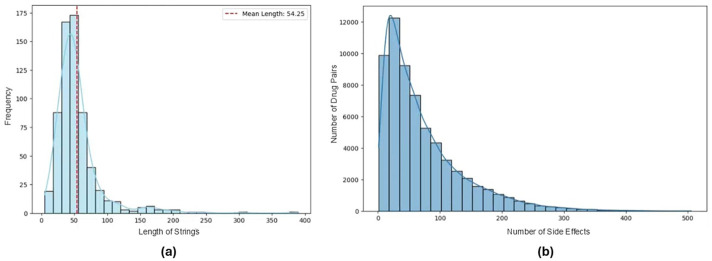
**(a)** Distribution of SMILES string lengths for 645 drugs in the dataset. **(b)** Number of side effects associated with each drug pair in the reformatted dataset.

To determine an optimal maximum input length, we analyzed the lengths of SMILES strings for all 645 drugs in the dataset. The average length was found to be 54.25 characters, as illustrated in Figure 1a. Based on this analysis, we set the maximum input length of language models to 64, ensuring that the majority of SMILES strings are preserved.

Furthermore, since we reformatted the dataset into a multi-label classification setup, it was essential to examine the sparsity within the side effect lists. Figure 1b reveals a highly skewed distribution in the number of side effects per drug pair, with a median of 52 and a mean of 72. This analysis highlights a significant class imbalance within the dataset, showing the unequal representation of side effects across drug pairs in this transformed format.

### 2.4 Embedding generation

To represent the SMILES strings of drugs in a format suitable for deep learning models, we use various language models to encode these text-based representations. This encoding maps SMILES strings into a latent space where critical structural and chemical information is preserved for downstream tasks.

In recent years, LLMs have played a pivotal role in various NLP tasks, including text analysis and translation. Their application in bioinformatics is expanding rapidly Lv et al. (2021), showcasing their versatility across diverse fields. In this study, we employ multiple models to encode drugs representations, including BERT, Sentence-BERT (SBERT) Reimers and Gurevych (2019), Fine-tuned ChemBERTa Xu et al. (2023), OpenAI’s GPT, Mol2vec Jaeger et al. (2018), and Doc2vec Le and Mikolov (2014).

BERT, built upon the transformer architecture, uses self-attention mechanisms to capture contextual relationships among words in a sequence by processing text bidirectionally. The BERT base model consists of 12 Transformer layers and 12 self-attention heads. SBERT extends BERT by adding a pooling layer to generate fixed-sized, sentence-level embeddings. In this study, we use base BERT (bert-base-uncased) for vectorizing both SMILES strings and side effect names.

In the context of chemical data, specialized language models such as ChemBERTa, or BioBERT Chithrananda et al. (2020); Lee et al. (2020) have been developed to effectively process chemical data, particularly SMILES strings. ChemBERTa, for instance, is tailored to enhance molecular property prediction. Variants of ChemBERTa differ primarily in their training objectives and the datasets used during pre-training. In this study, we use the ChemBERTa model pre-trained on the Masked Language Modeling (MLM) task using a dataset size of 77 million samples (ChemBERTa-77M-MLM). Additionally, we employ a fine-tuned variant of ChemBERTa developed by Xu et al. (2023), which uses SimCSE, a contrastive learning approach. This fine-tuning process is conducted using the GuacaMol benchmark dataset Brown et al. (2019), which serves as both the dataset and the basis for applying SimCSE to learn self-similarity in SMILES strings, with dropout introduced as noise to further enhance embedding quality.

For comparison with traditional models, we include Doc2vec, which extends Word2vec Mikolov et al. (2013) to encode larger text units, such as sentences. Additionally, Mol2vec is included as it specializes in substructure-based embeddings, and is trained on extensive molecular datasets like ZINC Irwin et al. (2012) and ChEMBL Gaulton et al. (2012); Bento et al. (2014) databases, which are well-established repositories of chemical compounds and bioactive molecules. Finally, to benchmark against state-of-the-art models, we assess the embeddings generated by OpenAI’s Third-generation GPT Small model (text-embedding-3-small). This provides a comprehensive comparison between traditional and advanced approaches.

Each model typically employs a distinct tokenization strategy. Tokenization involves segmenting input data into individual characters or substructures, depending on the model’s purpose. To compare the tokenization approaches of various language models, we consider 2-Mercaptoethanesulfonic Acid Sodium, represented by the molecular formula $C_{2}H_{6}O_{3}S_{2}$ . The corresponding SMILES notation is ”C(CS(=O)(=O)O)S”. This SMILES sequence was processed using the tokenizers of different models. Table 1 lists the tokenization results obtained from these models. Notably, the ChemBERTa models correctly identify ”C” and ”S” atoms as separate tokens, demonstrating their alignment with chemical structure conventions.

**TABLE 1:** Tokenization strategies of various language models for 2-Mercaptoethanesulfonic Acid Sodium $\left(C_{2}H_{6}O_{3}S_{2}\right)$ using the SMILES notation ”C(CS(=O)(=O)O)S”.

Model	Tokenization
BERT	[’C’, ’(’, ’CS’, ’(’, ’ = ’, ’O’, ’)’, ’(’, ’ = ’, ’O’, ’)’, ’O’, ’)’, ’S’]
SBERT	[’C’, ’(’, ’CS’, ’(’, ’ = ’, ’O’, ’)’, ’(’, ’ = ’, ’O’, ’)’, ’O’, ’)’, ’S’]
Deepchem ChemBERTa	[’C’, ’(’, ’C’, ’S’, ’(’, ’ = ’, ’O’, ’)’, ’(’, ’ = ’, ’O’, ’)’, ’O’, ’)’, ’S’]
Fine-tuned ChemBERTa	[’C’, ’(’, ’C’, ’S’, ’(’, ’ = ’, ’O’, ’)’, ’(’, ’ = ’, ’O’, ’)’, ’O’, ’)’, ’S’]
GPT	[’C’, ’(’, ’CS’, ’(’, ’ = ’, ’O’, ’)(’, ’ = ’, ’O’, ’)’, ’O’, ’)’, ’S’]

**FIGURE 2:**
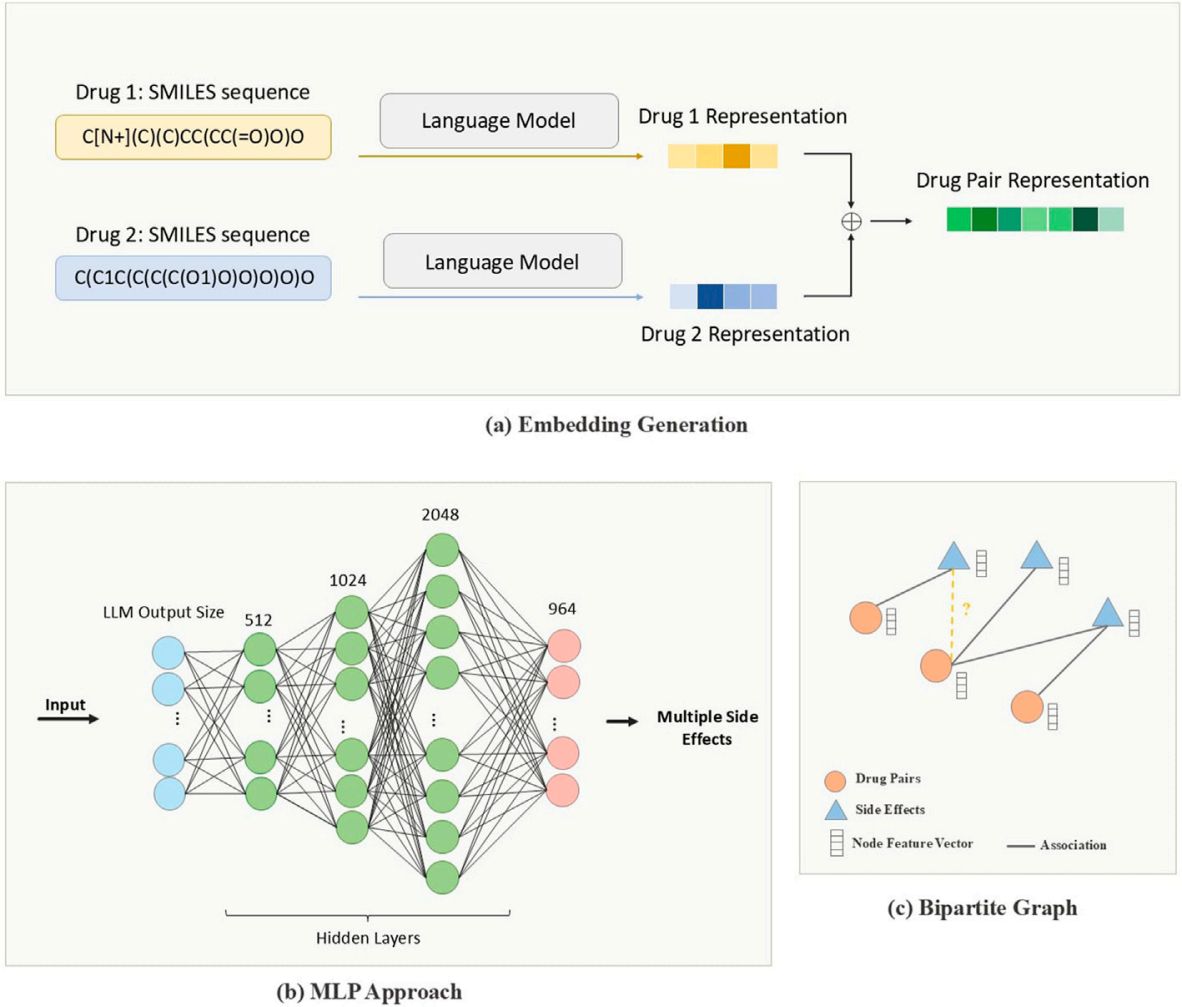
Pipeline of PolyLLM. **(a)** Drug-pair representations created by combining encoded drug features using language models **(b)** MLP approach: Multilayer perceptron to predict side effects from drug-pair representations. **(c)** Graph approach: Bipartite Graph constructed with drug-pair nodes and encoded side-effect features to predict whether or not a drug-pair is associated with side effects, considered as a binary link prediction task.

The vectorization process involves multiple steps, as illustrated in Figure 2a. Initially, each SMILES string is tokenized into individual characters or substructures, depending on the model specifications. Then, for BERT, SBERT, and ChemBERTa the tokenized SMILES strings are fed into the model to generate embeddings that capture rich contextual information. Mol2vec, by contrast, produces substructure-based embeddings that emphasize local chemical environments. Additionally, we use the Application Programming Interface (API) provided by OpenAI to encode drug representations using their advanced and most powerful third-generation embedding model. The *text-embedding-3-small* version, with an embedding dimension of 1,536, yields a compact informative representation per drug, resulting in a fused drug-pair embedding of the same dimensionality.

In our analysis, we evaluate four distinct strategies to obtain a comprehensive representation for each drug pair. Our experiments showed that both concatenation and summation yielded similar performance. However, to optimize computational efficiency, we selected summation as our fusion method. Specifically, after vectorizing each drug individually, we sum the embeddings of the two interacting drugs to produce a unified vector representation for each drug pair. Formally, given $e_{1}$ and $e_{2}$ as the embeddings of Drug-1 and Drug-2, respectively, the resulting drug-pair embedding, which serves as the input to our downstream models for predicting polypharmacy side effects, is represented as $e_{\mathrm{pair}}=\left[e_{1}+e_{2}\right]$ .

### 2.5 Classifier architecture

Given the embeddings generated, which provide structured numerical representations for each drug pair, we propose two distinct classification models to predict the side effects of polypharmacy.

The first model, illustrated in Figure 2b, is an MLP that, processes drug-pair embeddings to generate a list of predicted side effects. Although this approach demonstrates certain levels of effectiveness, it also has notable limitations. Specifically, due to the dataset’s inherent imbalance, the MLP model tends to produce a high number of false positive predictions. These limitations are discussed in more detail in subsequent sections. To address these limitations, we present an enhanced model based on GNNs.

**FIGURE 3:**
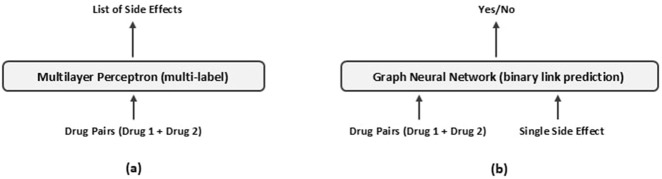
Overview of **(a)** the Multilayer Perceptron and **(b)** the Graph Neural Network approaches for predicting drug-pair side effects.

The GNN model, illustrated in Figure 2c, organizes the data into a graph-based structure, enabling it to capture complex relational patterns between drug pairs and their associated side effects. Unlike the MLP, this approach models the interactions more explicitly, aiming to predict the associations between each pair of drugs and potential side effects. Figure 3 illustrates the inputs and outputs of both classification models. In the following sections, we provide a detailed explanation of the architecture and methodologies for both models.

#### 2.5.1 Multilayer perceptron approach

We began by implementing an MLP, a fully connected feedforward neural network, as the initial classification model tailored to our dataset. The input to this model comprises the summed embeddings generated from two LLM components, providing a comprehensive numerical representation of each drug pair. The model’s output layer contains 964 neurons with sigmoid units, each representing the probability of a specific side effect occurring for the given drug combination.

After extensive experimentation to optimize the architecture, as outlined in Table 2, we determined that a design with three hidden layers of dimensions of 512, 1,024, and 2048, effectively captures the complex features and relationships encoded in the SMILES representations.

**TABLE 2:** AUC scores of the MLP approach with varying numbers of layers and neurons.

Number of layers	Neurons per layer	AUC
1	512	0.854
1	1,024	0.861
1	2,048	0.863
2	256, 512	0.866
2	512, 1,024	0.878
2	1,024, 2,048	0.882
3	128, 256, 512	0.857
3	256, 512, 1,024	0.880
3	512, 1,024, 2,048	**0.886**

Note: The best performance is highlighted in bold.

Additionally, we evaluated several activation functions and selected the Leaky Rectified Linear Unit (Leaky ReLU) Xu et al. (2015) with a negative slope of 0.1 for each hidden layer. Leaky ReLU was selected for its ability to address the vanishing gradient problem, which is a common issue in activations functions such as tanh or sigmoid , and also for its capacity to enhance learning stability.

To further stabilize the learning process, batch normalization Ioffe and Szegedy (2015) was applied after the first hidden layer. This step was critical as the initial layer of the neural network handles raw input data, which may vary widely in distribution. However, experimentation with batch normalization on other layers indicated a slight performance decrease, potentially due to disruptions in the natural distribution of intermediate representations.

To mitigate overfitting, we added dropout Srivastava et al. (2014) with a rate of 0.2 in each hidden layer. This dropout rate was selected after comprehensive testing with various dropout values, as summarized in Table 3, to ensure an optimal balance between the model’s generalizability and its performance.

**TABLE 3:** AUC results of the MLP approach across different hyperparameters.

Dropout	Learning rate		
	0.001	0.005	0.0001
0.1	0.878	0.880	0.854
0.2	0.881	**0.882**	0.855
0.3	0.880	**0.881**	0.852
0.4	0.876	0.877	0.848
0.5	0.869	0.868	0.840

Note: The best performance is highlighted in bold.

#### 2.5.2 Bipartite graph

To address the limitations of the MLP approach, discussed in subsequent sections, we introduced a graph-based classification model that mitigates the challenges of data imbalance.

We transform the drug pairs and their associated polypharmacy side effects into a graph structure to capture their real-world relationships. By focusing on the relational nature of the problem, the task can be reformulated as link prediction over the data. The graph is defined as G=(D,S,E,F) , where $D={\left\{d_{i}\right\}}_{i=1}^{n}$ represents the drug-pair set, $S={\left\{s_{j}\right\}}_{j=1}^{m}$ denotes the polypharmacy side effect set, $E=\left\{d_{i}s_{j}|d_{i}\in D,s_{j}\in S\right\}$ is the set of edges representing associations between drug-pairs and side effects, and $F=\left\{f_{d_{i}},f_{s_{j}}\right\}$ represents the feature set for the nodes. Specifically, $f_{d_{i}}$ corresponds to the embeddings of drug pairs generated by language models, while $f_{s_{j}}$ corresponds to the BERT-based embeddings of side effect names. This formulation emphasizes the bipartite nature of the graph, since connections exist only between drug-pair nodes and side effect nodes, with no direct edges within D or S .

To construct the graph, we used the original dataset format, where each row represents the relationship between a drug pair and a single side effect. As a result, this graph contains 63,472 drug pair nodes, 964 side effect nodes, and 4,576,785 edges. This defines the task as a binary link prediction problem, where the model learns to predict whether an association exists between a drug pair and a side effect.

Each node is assigned a unique identifier derived from the corresponding dataframe and is equipped with features reflecting its characteristics. More precisely, drug-pair nodes are assigned features derived from molecular embeddings, while side effect nodes use features extracted from vectorizing their names. By combining these structural identities with node features, the model captures the topological relationships and the contextual information of the nodes. This approach allows the GNN to better differentiate between nodes.

To achieve this, node IDs are first mapped into a latent space of size 64 and are then combined with the precomputed features of drug pairs and side effects, derived from language models. Additionally, it is essential to ensure that all node features have identical dimensions for effective model training. Currently, we use BERT to vectorize the names of side effects, which generates embeddings with a size of 768. For drug pairs, we experiment with various language models, each producing embeddings of different dimensions. For instance, Mol2Vec generates embeddings of size 300. Therefore, we project both the drug pair features and the side effect features into a 64-dimensional space using a simple one-layer neural network to achieve consistency in dimensionality across all nodes.

To have a trainable module, we design a graph neural network to operate over graph G . This graph neural network has two major parts:

• Encoder: Responsible for learning node embeddings by aggregating information from graph G .

• Decoder: Predicts the likelihood of an edge between a drug pair and a side effect based on their learned embeddings.

##### 2.5.2.1 Encoder

The graph encoder model takes graph G as input, along with the node features $f_{d_{i}}$ and $f_{s_{j}}$ and generates a d -dimensional embedding $z_{i}\in R^{d}$ for each node. The precomputed drug-pair features and side effects features are set as the initial embedding values for their respective nodes. By initializing nodes with informative features, the model can learn richer embeddings by propagating and aggregating information from neighboring nodes and the node itself. The impact of using these initial embedding values is investigated in the Results section.

The graph convolutional layer used in this study is the GraphConv Morris et al. (2018) layer from the PyTorch Geometric library. For a given node, the convolutional layer aggregates features from its neighboring nodes, scales them with a learnable weight parameter, and adds them. On the other hand, the node’s own feature is also multiplied by another learnable weight parameter. These contributions are then summed to produce the updated feature for the node. Mathematically, a single layer of this model is expressed in Equation 1.

$$h_{i}^{k}=W_{1}^{\left(k\right)}h_{i}^{k-1}+W_{2}^{\left(k\right)}\underset{j\in N\left(i\right)}{\sum }h_{j}^{\left(k-1\right)}$$ (1)

Here, $h_{i}^{k-1}$ is the feature vector of node i at the (k−1) -th layer, $h_{i}^{k}$ is the updated feature vector of node i at the k -th layer, $W_{1}^{\left(k\right)}$ and $W_{2}^{\left(k\right)}$ are learnable weight matrices for self-connections and neighboring node features, respectively, and N(i) denotes the set of neighbors of node i .

Once the message passing process is completed, resulting in finalized node features, it is important to note that the current graph initially contains only positive edges, representing associations between $d_{i}$ and $s_{j}$ . However, for the model to effectively distinguish between associated and unassociated drug pairs and side effects, negative edges are introduced. To achieve this, for every positive edge, a negative edge is added by first excluding the already-connected node pairs and then randomly selecting other node pairs to be labeled as zero. This is done by using utilities provided by the PyTorch Geometric library. Consequently, each mini-batch contains both positive and negative edges, allowing the model to learn to differentiate between the two during training.

##### 2.5.2.2 Decoder

As discussed, during the encoder process, node embeddings are iteratively learned and updated to capture the properties of the graph. In the decoder process, the objective is to compute a function $f\left(d_{i},s_{j}\right)$ that estimates the likelihood of an association between drug $d_{i}$ and side effect $s_{j}$ . To achieve this, the decoder uses the learned embeddings of nodes $d_{i}$ and $s_{j}$ , denoted as $h_{d_{i}}^{\left(k\right)}$ and $h_{s_{j}}^{\left(k\right)}$ , where k represents the final layer of the encoder. We used the dot product in the decoder , as shown in Equation 2, to calculate the score $s_{ij}$ which represents the association likelihood.

$$s_{ij}=h_{i}^{\left(k\right)}.h_{j}^{\left(k\right)}$$ (2)

This score $s_{ij}$ directly reflects the correlation between the embeddings and provides a scalar score as the likelihood.

### 2.6 Training the model

In the following sections, we provide a detailed, separate discussion on the training procedures of two classifiers.

#### 2.6.1 Multilayer perceptron

The drug pairs, along with their associated side effects, are partitioned into training, validation, and test sets. To ensure that our results are comparable with baseline studies, the dataset is divided as follows: 80% for training, 10% for validation, and 10% for testing. Comprehensive measures were taken to ensure no data leakage occurred between the test set and the other subsets. Following this, 10-fold cross-validation was performed on the training and validation sets to further evaluate the model’s robustness.

This classification model was trained using the drug-pair embeddings generated by each language model for up to 100 epochs, with early stopping used to prevent overfitting during the training process.

To address the issue of class imbalance to some extent, Binary Focal Cross Entropy Lin et al. (2017) was chosen as the loss function, as it places greater emphasis on hard-to-classify samples, offering an advantage over standard Binary Cross Entropy. Furthermore, an exponential decay learning rate schedule with a decay rate of 0.96 was applied to progressively reduce the learning rate during training, helping the model converge more effectively.

#### 2.6.2 Bipartite graph

To facilitate message exchange across all nodes in the graph, we transform the graph into an undirected structure. This is achieved by introducing reversed edges for every existing edge solely during the message-passing phase, ensuring that relationships are preserved irrespective of directionality. This transformation doubles the size of the graph, expanding it from 4,576,785 to 9,153,570 edges. In the decoder phase, the model focus is only on the existence or absence of associations, disregarding the directionality of edges.

To prepare the dataset for this graph construction and ensure compatibility with other baselines, the data is partitioned into three subsets: 80% for training, 10% for validation, and 10% for testing. It is important to note that a transductive approach is employed for edge splitting. Specifically, only 30% of the training edges are considered for prediction (supervision), loss computation, and model weight updates, while the remaining 70% are reserved for message passing. This selection was made after trying other ratios, including 90/10, 60/40, 30/70, and 10/90 splits (training/message passing). We observed that extreme configurations (e.g., 90/10 or 10/90) resulted in performance degradation, likely due to insufficient supervision or lack of connectivity, respectively. In contrast, 60/40 and 30/70 produced comparable results, and we selected 30/70 for our final setup. During validation, all training edges, including those used for supervision and message passing, are used to predict validation edges. Similarly, in the testing phase, the model uses all training and validation edges to predict test edges.

Considering the binary nature of the link prediction task, we opted for Binary Cross Entropy With Logits, a well-suited loss function for binary classification tasks. This choice is particularly advantageous as the final layer of the model outputs raw logits directly. By combining a Sigmoid layer and Binary Cross Entropy Loss into a single class, this loss function offers numerical stability and computational efficiency compared to using a standalone Sigmoid layer followed by BCELoss. Additionally, to mitigate the risk of overfitting, we implemented the Early Stopping technique, which stops training when validation performance plateaus.

## 3 Experiments

### 3.1 System configuration

We conducted all the experiments on a high-performance computing system equipped with an Intel(R) Xeon(R) Platinum 8,480+ CPU, and four NVIDIA H100 SXM5 GPUs, each with 80 GB of memory. For implementation, we used Python 3.11.2 and Scikit-learn 1.4.0 for both the MLP and GNN models. Additionally, we utilized Keras 2.15.0 for the MLP model and PyTorch 2.4.1 with PyTorch Geometric 2.6.1 for the GNN model, along with other relevant libraries.

### 3.2 Baselines

In this section, we introduce some of the well-known studies in polypharmacy side effect prediction to compare their performance with our proposed models.

• *Decagon* Zitnik et al. (2018) The Decagon paper introduces a multimodal graph with various node types, including drugs and proteins. Each drug-drug interaction is labeled by the associated side effect. They then apply a graph convolutional neural network (GCN) for the multi-relational link prediction in the multimodal network.

• *RESCAL tensor decomposition* Nickel et al. (2011) This approach employs a tensor factorization built on a multi-relational structure. $M_{i}$ which represents a drug-drug association for drug pairs with side effect r is defined as $M_{r}=AT_{r}A^{T}$ for 964 different side effects ( $T_{r}$ and A are model parameters). $a_{i}T_{r}a_{j}$ also defines the predicted association of drugs i and j with side effect type r .

• *DEDICOM tensor decomposition* Papalexakis et al. (2017) This approach is also based on tensor factorization, suitable for sparse data settings. Given $M_{i}$ as a drug-drug matrix, it can be decomposed as $M_{r}=AU_{r}TU_{r}A^{T}$ . The association of pair of drug i and j with associated side effect r is defined as $a_{i}U_{r}TU_{r}a_{j}$ .

• *DeepWalk neural embeddings* Perozzi et al. (2014) In this approach, nodes learn d-dimensional neural features through a biased random walk procedure that explores network neighborhoods of nodes. The drug pairs are represented by concatenating the representation of the drugs and are fed into a logistic regression classifier. Separate logistic regression classifiers are trained for different link types (i.e., side effect types).

• *Concatenated drug features* Zitnik et al. (2018) This approach creates a feature for each drug by applying PCA to both the drug-target protein interaction matrix and the side effects of individual drugs. The feature vectors of each drug are then concatenated to represent a drug pair. The drug pairs are fed into gradient boosting trees classifier, which predicts the associated side effect of that drug pair.

### 3.3 Performance metrics

In this section, we analyze the performance metrics that are used to compare our proposed models with existing baselines. We use three widely adopted performance metrics: Area Under the Receiver Operating Characteristic Curve (AUC-ROC), the Area Under the Precision-Recall Curve (AUPRC), and the Average Precision at 50 (AP@50). These metrics are chosen to align with the evaluation frameworks used in prior studies, ensuring a fair and consistent comparison.

The ROC curve addresses the challenges of finding the optimal threshold to classify probabilities by computing the True Positive Rate (TPR) and False Positive Rate (FPR) at various thresholds. The AUC-ROC metric, which is the area under the ROC curve, is defined in Equation 3.

$$\text{AUC}=\int_{0}^{1}\text{TPR}\left(x\right)\;\text{dFPR}\left(x\right)$$ (3)

On the other hand, the Precision-Recall Curve plots Precision against Recall at different thresholds. The AUPRC metric, which represents the area under this curve, is computed defined in Equation 4.

$$\text{AUPRC}=\int_{0}^{1}\text{Precision}\left(r\right)\;\text{dRecall}\left(r\right)$$ (4)

When dealing with ranked predictions, Average Precision at k (AP@ k ) is particularly useful for evaluating the quality of the top k predictions. In real-world clinical scenarios, healthcare experts often prioritize the most critical and probable side effects due to time and resource constraints. Therefore, ranking model predictions is a crucial aspect for practical applicability in healthcare. To compute AP@ k , predictions are first ordered by their confidence scores, and precision is calculated separately for the top k elements, as shown in (Equation 5a). The average of these precision values is then taken, as illustrated in (Equation 5b).

$${AP}_{k}=\frac{\text{Number of true positives in top }k\text{ predictions}}{k}$$ (5a)

$$AP@50=\frac{1}{n}\overset{n}{\underset{k=1}{\sum }}{AP}_{k}$$ (5b)

AUC-ROC vs. AUPRC It is commonly believed that AUC-ROC is inflated in imbalanced datasets, and AUPRC is considered a more reliable indicator of model performance in such cases. However, several studies Richardson et al. (2024); McDermott et al. (2024) have demonstrated that this is a misinterpretation of the differences between the ROC and PR curves. These studies show that AUC-ROC is not significantly affected by class imbalance and provides a more robust and invariant measure of performance, even in imbalanced datasets Richardson et al. (2024). In contrast, they show that AUPRC is highly sensitive to class imbalance and changes drastically depending on the dataset’s class distribution. In other words, AUC-ROC provides a general estimate of classifier performance, while AUPRC reflects the classifier’s performance on a specific dataset. Reference Richardson et al. (2024) recommends using AUC-ROC alongside AUPRC and AP@50, as it enables fairer comparisons of models across datasets with varying class distributions.

In clinical contexts, rare events are often of primary interest. A recent study by Martin et al. (2025) highlights that in highly imbalanced datasets, such as those involving critical illness events, AUROC can be misleadingly high due to its dependence on true negative rates, which often dominate in rare-event settings. In contrast, AUPRC focuses on the tradeoff between precision and recall, which are more operationally relevant in clinical decision-making. The authors argue that AUPRC better reflects a model’s ability to reliably detect rare outcomes while avoiding excessive false positives, which can lead to diminished trust in predictive models. In our work, where the goal is to identify clinically significant polypharmacy side effects, we adopt AUPRC alongside AUROC to more accurately capture model performance under real-world, imbalanced conditions.

### 3.4 Fusion evaluation

**FIGURE 4:**
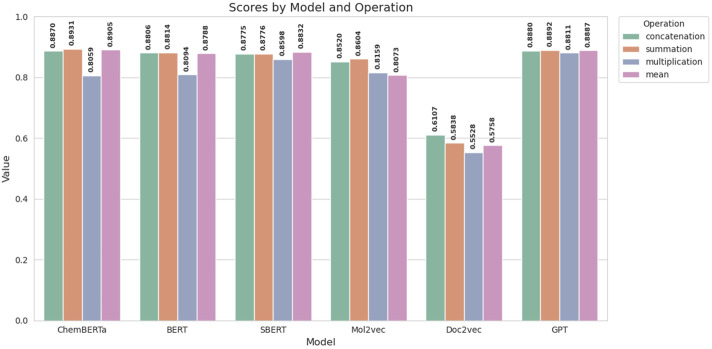
AUC scores for various fusion strategies across multiple encoders.

There are several ways to fuse two entities, and the choice of fusion strategy plays a critical role in determining model performance. In this section, we explore several fusion strategies, including concatenation, summation, multiplication, and mean, to merge two drug representations. As shown in Figure 4, these strategies have been evaluated across various encoders. The results demonstrate that both summation and concatenation effectively capture the necessary information, though concatenation tends to increase the computational complexity due to the larger resulting embedding size. Conversely, while the mean strategy is generally effective, it may lead to information loss and underperformance in certain scenarios. Given the comparable performance of summation and concatenation, we opt for summation as our fusing method to minimize computational overhead while maintaining model efficacy.

### 3.5 Hyperparameter setting

#### 3.5.1 MLP approach

We conducted a thorough investigation into the impact of key hyperparameters, such as learning rate and dropout rate, on the model’s performance. This is a critical analysis as it ensures the model’s ability to generalize effectively and avoid overfitting. The AUC scores obtained by varying the learning rate across different dropout rates are presented in Table 3. Notably, the model achieves the highest AUC score of 0.882 with a learning rate of 0.005 and a dropout rate of 0.2.

Additionally, we evaluate the performance of neural networks with varying numbers of hidden layers and neurons per layer. As shown in Table 2, the model with three hidden layers, comprising 512, 1,024, and 2048 neurons respectively, achieves the best AUC score of 0.886 across all configurations.

To ensure that the observed improvements in AUC scores were specifically attributable to the different regularization techniques applied, various configurations were tested individually and in combination, as detailed in Table 4. Ultimately, the combination of Dropout and Batch Normalization was chosen, as it demonstrated the most effective performance.

**TABLE 4:** AUC scores of the MLP approach under different regularization configurations.

Regularization	AUC
None	0.873
Dropout	0.891
Batch Normalization	0.884
Dropout + Batch Normalization	**0.893**

Note: The best performance is highlighted in bold.

#### 3.5.2 Graph approach

We analyzed the impact of the learning rate on the model performance. The optimal learning rate was determined empirically by testing multiple values, including 0.1, 0.5, and 0.05. The results indicated that a learning rate of 0.01 yielded the best performance.

Additionally, dropout rates of 0.1, 0.3, 0.5, 0.7, 0.8, and 0.9 were manually tested, and a value of 0.8 was selected. This choice accounts for the dataset’s large size and helps mitigate overfitting by preventing the model from becoming overly reliant on specific training examples.

## 4 Results and discussion

### 4.1 Comparison of models

This section discusses the results of our experimental studies on the Decagon dataset, evaluating 964 polypharmacy side effects and comparing the proposed models with each other as well as with alternative approaches.

The average AUROC, AUPRC, and AP@50 scores, along with their standard deviations for the MLP classifier, are reported in table 5. Across 964 side effect types, transformer-based language models achieved competitive AUC scores compared to prior studies and traditional methods such as Mol2vec and Doc2vec. In particular, the fine-tuned ChemBERTa model, which is pre-trained and fine-tuned on SMILES strings, achieved the highest AUC score of 0.8894 and AP@50 score of 0.8123, both surpassing baseline scores. However, all models showed relatively low AUPRC scores, suggesting a tendency to predict false positives. This limitation can be attributed to the dataset’s high class imbalance. As Richardson et al. (2024); McDermott et al. (2024) noted, while the AUC-ROC score is a strong indicator of the overall performance of the model, the AUPRC reflects the performance of the model in a specific dataset. Therefore, despite the challenges of false positives, the fine-tuned ChemBERTa model demonstrates strong overall performance based on its high AUROC and AP@50 scores.

**TABLE 5:** Performance of integrating language model features with the bipartite graph compared to baseline methods.

Model	AUC	AUPRC	AP@50
Baselines			
RESCAL	0.693	0.613	0.476
DEDICOM	0.705	0.637	0.567
DeepWalk neural embeddings	0.761	0.737	0.658
Concatenated features	0.793	0.764	0.712
Decagon	0.872	0.832	0.803
MLP Approach			
ChemBERTa SimCSE	0.8894±0.0017	0.4139±0.0081	0.8123±0.0125
Deepchem ChemBERTa	0.8859±0.0019	0.3979±0.0079	0.7557±0.0120
GPT (small)	0.8886±0.0039	0.4129±0.0166	0.7820±0.0218
BERT	0.8793±0.0034	0.3736±0.0119	0.7157±0.0182
SBERT	0.8717±0.0044	0.3494±0.0121	0.6808±0.0169
Mol2vec	0.8498±0.0057	0.3044±0.0109	0.6266±0.0149
Doc2vec	0.8684±0.0032	0.3376±0.0091	0.6593±0.0173
GNN Approach			
ChemBERTa SimCSE	0.9221±0.0011	0.8929±0.0007	0.7693±0.0200
Deepchem ChemBERTa	0.9228±0.0039	0.8944±0.0025	0.9599±0.0044
GPT (small)	0.9181±0.0010	0.8900±0.0003	0.8640±0.0065
BERT	0.7951±0.0463	0.8238±0.0259	0.8879±0.0084
SBERT	0.6745±0.0040	0.7141±0.0029	0.8170±0.0094
Mol2vec	0.8382±0.0682	0.8152±0.0604	0.8144±0.0472
Doc2vec	0.7273±0.0174	0.7519±0.0128	0.5146±0.0555
Zeros	0.0740±0.0004	0.3355±0.0000	0.0000±0.0000

The results show that the fine-tuned ChemBERTa is capable of capturing token-level chemical information and excelling in modeling complex drug pair interactions by leveraging attention mechanisms to account for long-range dependencies. Among the models evaluated, only GPT, which is trained on a vast and diverse amount of data, achieves results comparable to ChemBERTa. Other transformer-based models, such as BERT and SBERT, also outperform Decagon in terms of AUC score, showcasing their ability to capture intra-molecular and inter-molecular relationships. However, traditional models like Mol2vec and Doc2vec continue to struggle to achieve competitive performance.

Reframing the problem into a graph-based approach and training a graph neural network with drug-pair node features from various language models effectively mitigated the issue of class imbalance. As shown in Table 5, Deepchem ChemBERTa achieved an impressive AUC score of 0.92 and AUPRC of 0.89 outperforming baseline studies. Additionally, the Fine-tuned ChemBERTa produced results very close to Deepchem ChemBERTa, suggesting that while fine-tuning does not degrade performance, it may not necessarily yield significant improvements either. The strong performance of both ChemBERTa models can be attributed to their pre-training on chemical data, which both were trained on 77 million unique SMILES from PubChem, making them well-suited for this context. Also, as table 1 illustrated, the tokenization strategy of the ChemBERTa model, called Bye-Pair Encoder (BPE) Wolf et al. (2019), effectively tokenizes atoms, while models like BERT and SBERT struggle to separate Carbon from Sulfur.

GPT embeddings also demonstrate highly competitive performance, achieving an AUC score of 0.9181 and AUPRC of 0.8944. Despite GPT not being specifically trained on chemical data, its extensive pre-training on diverse datasets contributes to its strong performance, making it comparable to ChemBERTa. Even though Table 1 shows that the tokenization strategy of GPT struggles to distinguish certain chemical elements, the model appears to compensate by using its pre-trained knowledge.

Mol2vec, with an AUC of 0.8382 and AUPRC of 0.8152, outperforms Doc2vec but still lags behind ChemBERTa and GPT-based embeddings. This is likely because it is specifically trained and designed for molecular representation and captures the structural properties of drugs. However, since it is trained in an unsupervised manner, it may not encode interactions as effectively as transformed-based models tailored for chemical data. Doc2vec, with a low AUC score of 0.7273, indicates that traditional models lacking attention mechanisms and domain-specific training may not be suitable as standalone approaches in this context.

SBERT, built on top of BERT, treats drug pair representations as sentences and applies pooling to generate a single embedding per drug pair. However, it seems that this pooling strategy led to a loss of critical molecular features, making it harder for the model to capture structural details. As a result, SBERT performs poorly compared to both BERT and even Doc2vec, despite having an attention mechanism. Comparing BERT and SBERT in the GNN setting reveals that since BERT preserves token-level embeddings, it can retain more detailed molecular information, while SBERT’s pooling mechanism limits the GNN’s ability to capture atomic interactions and meaningful relationships.

We also trained the GNN model with zero-filled features (size 200) to evaluate the impact of informative and meaningful embeddings. The results show that zero features performed the worst, with an AUC of 0.07, confirming the necessity of meaningful representations.

The Decagon model, which uses Graph Convolutional Networks for drug-drug, drug-protein, and protein-protein interactions, is primarily limited by fixed node embeddings and does not leverage transformer-based representations. Our approach benefits from pre-trained ChemBERTa embeddings that capture richer chemical information and improve generalization. Additionally, while Decagon integrates other biological entities such as proteins, we rely solely on chemical structures as features. Given this, our model achieves competitive results, demonstrating the effectiveness of transformer-based architectures even with a limited feature set. Furthermore, in our approach, drug pairs are explicitly modeled as a distinct node type since side effects arise due to specific drug combinations, helping capture side-effect-specific interaction patterns more effectively than a node-centric approach.

While our approach uses the chemical structures of drugs alone through SMILES-based LLM embeddings, we acknowledge that many adverse drug reactions are mediated by complex biological mechanisms that cannot be fully captured by molecular structure alone, as further discussed in the Side Effect Analysis section. Biological factors such as protein interactions, metabolic pathways, pharmacogenomics, and off-target effects may play crucial roles in this context. Several prior studies have incorporated protein interactions Masumshah et al. (2021), drug pathways, and enzymes Masumshah and Eslahchi (2023) to better capture these mechanistic pathways, potentially enhancing predictive power and interpretability. In the present study, our primary focus was to analyze drug entities alone in order to better explore the applicability of LLMs for polypharmacy side effect prediction. As LLM-based approaches have not yet been extensively applied to this task, we intentionally limited the scope to chemical structures to avoid additional complexities in this initial investigation. In future work, we plan to extend the model by incorporating additional biological entities such as proteins, pathways, and enzymes to capture more complex mechanistic interactions.

### 4.2 Side effect analysis

We obtained the best and worst predicted side effects by the Deepchem ChemBERTa model integrated with the bipartite graph. The results are shown in Table 6, which is ordered by AUPRC scores. The results show that GNN performs best on side effects with strong molecular underpinnings, likely because these side effects are directly influenced by drug interactions at a biochemical level, making them easier for the model to capture using molecular-based embeddings. The analysis of the best and worst-performing side effects in the table highlights that side effects with a significantly lower number of drug-drug interactions in the dataset tend to have lower AUPRC scores. For instance, bronchiolitis, which appears to be the worst-performing side effect, is linked to only 636 drug interactions, whereas cardiac decompensation, the best-performing side effect, has 16,555 interactions. This discrepancy in data availability plays a major role in the model’s predictive performance.

**TABLE 6:** Side effects showing the best and worst performance when DeepChem ChemBERTa features are integrated with the bipartite graph.

Best performing side effects	AUROC	AUPRC	Worst performing side effects	AUROC	AUPRC
Cardiac decompensation	0.988177	0.996100	Bronchiolitis	0.949642	0.613721
Cardiac ischemia	0.986724	0.993551	Corneal ulcer	0.925869	0.623843
Aching joints	0.974858	0.992379	Alcoholic intoxication	0.880503	0.625518
Abnormal LFTs	0.980251	0.991889	Aseptic meningitis	0.923545	0.636184
Acne	0.990405	0.991439	Phimosis	0.954801	0.643596
Burns Second Degree	0.996599	0.987317	Primary biliary cirrhosis	0.958360	0.656204
Acidosis	0.955826	0.986312	Brain abscess	0.953811	0.658020
Extremity pain	0.962125	0.984298	Bundle branch block	0.906469	0.658819
Abnormal Laboratory Findings	0.980074	0.984159	Fibrosing alveolitis	0.942627	0.667376
*Candida* Infection	0.964859	0.981878	SCLC	0.957948	0.668350

We also examined the worst-performing side effect in terms of AUC score, which happens to be Adenopathy, with an AUC of 0.7844 and an AUPRC of 0.8398. Adenopathy refers to swelling of lymph nodes, and as some studies Maini and Nagalli (2023) have shown, it is primarily caused by immune-mediated mechanisms or infections rather than direct chemical interactions between drugs. Since the ChemBERTa-derived features integrated into our graph are designed to capture primarily chemical interactions between drugs and are not equipped to model complex biological processes, they may only detect general patterns of drug interactions correlated with Adenopathy. To further investigate this limitation, we analyzed a specific drug combination in the dataset reported to cause Adenopathy: Cyclophosphamide and Gamma-Aminobutyric Acid (GABA). Cyclophosphamide is a well-known immunosuppressive drug Ogino and Tadi (2023); Winkelstein (1973); Ahlmann and Hempel (2016) and is widely used in chemotherapy and autoimmune conditions. GABA, on the other hand, is found to have immunomodulatory properties and can influence immune cell function Jin et al. (2013). When combined, these compounds may interact in a manner that amplifies or alters their individual immunomodulatory effects. This imbalance could trigger immune activation, potentially leading to adenopathy as a side effect. Given that the immunomodulatory effects of Cyclophosphamide and GABA are indirect and involve complex biological pathways, our model, which primarily focuses on direct chemical interactions between drugs, is likely unable to fully capture these intricate mechanisms.

## 5 Conclusion

The adverse effects associated with polypharmacy represent the primary challenges when patients are administered multiple drugs concurrently. Accurately identifying and predicting these side effects can provide valuable insights for clinicians. Most existing predictive methods for drug combinations rely on diverse data sources, such as cell lines and protein networks. In this study, we utilized a text-based molecular representation known as SMILES and encoded drug SMILES strings using various language models. By aggregating the representations of individual drugs, we obtained a single embedding representing each drug pair. These embeddings are then fed into two distinct classifiers including a multilayer perceptron and a graph neural network. Our results indicate that integrating ChemBERTa-derived features within a graph neural network setting yields the best performance, surpassing baseline models. Additionally, we demonstrated that using fewer features, such as chemical formulas, alongside informative embeddings can reduce the dependency on extensive biological data. In future work, we plan to enhance the model by incorporating additional biological entities such as proteins, pathways, and enzymes alongside chemical structures, as well as exploring robustness to multiple SMILES representations per drug, to improve its ability to capture complex relationships. Additionally, we plan to incorporate explainability into the model to improve interpretability, and to enhance its robustness by enabling it to handle less standardized inputs, such as synonym variations in side effect terminology.

## Data Availability

The dataset used for this study is available through the Decagon’s study website (https://snap.stanford.edu/decagon).The code for this study is available from the GitHub repository (https://github.com/sadrahkm/PolyLLM). Further inquiries can be directed to the corresponding author.
